# Case report: Successful treatment of refractory membranous nephropathy with telitacicept

**DOI:** 10.3389/fimmu.2023.1268929

**Published:** 2023-10-17

**Authors:** Lei Zhang, Hua Jin, Dong Wang, Yiping Wang

**Affiliations:** Department of Nephrology, The First Affiliated Hospital of Anhui University of Chinese Medicine, Hefei, China

**Keywords:** case report, successful, treatment, refractory membranous nephropathy, telitacicept

## Abstract

Despite various treatment methods, the remission rate of membranous nephropathy remains limited. Refractory membranous nephropathy especially lacks effective treatment plans. Telitacicept achieves comprehensive inhibition of CD20-positive B cells, plasma cells, and T cells, thereby bringing new hope to the treatment of membranous nephropathy and refractory membranous nephropathy. Here, we report a case of a 46-year-old man with membranous nephropathy. Although the combined treatment with glucocorticoid, tacrolimus, mycophenolate mofetil, cyclophosphamide, and rituximab was not successful, the patient achieved complete remission of urinary protein after glucocorticoid combined with telitacicept. This is the first report on the application of telitacicept in the treatment of membranous nephropathy, especially refractory membranous nephropathy. The application of telitacicept in the treatment of membranous nephropathy deserves further attention.

## 1 Introduction

The histomorphological substrate of membranous nephropathy (MN) is the accumulation of immune deposits in the subcutaneous space above the glomerular filtration barrier. Most frequently, MN presents as proteinuria in nephrotic range, with or without other elements of nephrotic syndrome. MN occurs in all regions and races, and it has an annual incidence rate of 10%–12% in North America and 2%–17% in Europe. The average age at diagnosis is 50–60 years, and the male-to-female ratio is 2:1. Spontaneous remission of untreated MN occurs in approximately one-third of patients, with a 10-year renal survival rate of 60%–80% (1). In the past decade, the understanding of the pathogenesis of MN has substantially improved. Unlike other autoimmune kidney diseases, the pathogenic circulating autoantibodies against M-type phospholipase A2 receptor (PLA2R1) and thrombospondin type-1 domain-containing 7A (THSD7A) are considered the main factors leading to MN (2). Regarding the treatment of MN, the Kidney Disease: Improving Global Outcomes (KDIGO) 2021 guidelines emphasize that appropriate treatment plans should be selected based on the clinical risk assessment of progressive loss of kidney function (3). The use of rituximab in the treatment of MN was first reported in 2002 (4). Relevant clinical studies of MN have confirmed that the remission rate of rituximab treatment is 57%–89% (5, 6), while that of glucocorticoid combined with cyclophosphamide (CYC) and that of glucocorticoid combined with tacrolimus are about 88% and 53%, respectively (7). However, regardless of the treatment plan, the remission rate remains limited. Determining a new treatment plan is a great challenge when the abovementioned drug treatments fail. Considering the pathogenesis of MN, compared with traditional CD20-targeting biological inhibitors, telitacicept realizes the overall inhibition and regulation of lymphocyte growth process, including plasma cells and T cells, greatly reducing the risk of circulating and *in situ* immune complex formation, thereby achieving therapeutic effects. This is the first report of complete remission of refractory MN in 24h proteinuria following telitacicept treatment.

## 2 Case report

During physical and biochemical examination in October 2019, a 46-year-old man showed edema of both lower limbs and urinary protein level of 3+. In November 2019, the serum albumin (SA) level was 28.9 g/L, 24h proteinuria was 2.87 g, and blood PLA2R and THSD7A were negative.

### 2.1 Kidney biopsy results

**Figure 1:**
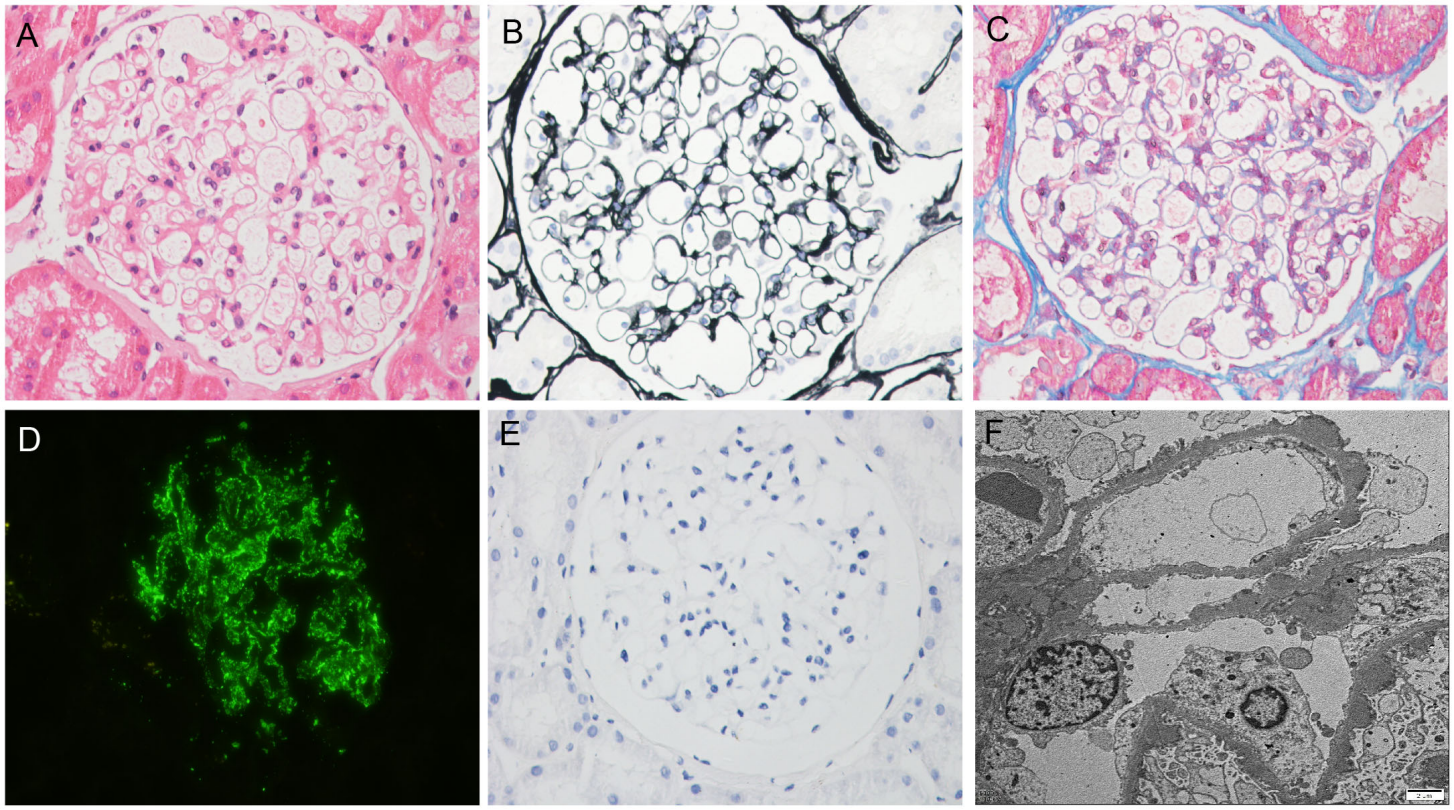
Biopsy findings. **(A)** Hematoxylin–eosin staining (×400); **(B)** Periodic-acid silver methenamine staining (×400); **(C)** Masson staining (×400); **(D)** Immunofluorescence staining for PLA2R (×400); **(E)** Immunohistochemical staining for THSD7A (×400); **(F)** Electron microscopy (×10000).

The results of immunofluorescence were as follows: IgG, ++++; IgA, +; IgM, +; C3, +++; C1q, -; Kappa, +++; Lambda, +++; IgG1, ++; IgG2, -; IgG3, -; and IgG4, +++. Light microscopy showed that the number of glomerular cells was slightly increased, the basement membrane was thickened in segments, nail process was occasionally formed, mesangial cells and matrix were slightly proliferated, and subepithelial eosinophils were deposited. Congo red staining was negative. Immunofluorescence of PLA2R was positive, while immunohistochemistry of THSD7A was negative. Electron microscopic examination showed the following results. The basement membrane was irregularly thickened (the thickness of the thin part was about 240 nm–300 nm, the thickness of many parts was about 380 nm–700 nm, and the thickness of the thickest part was 1300 nm), and the foot processes of podocytes were diffusely fused (> 90%). A large amount of electron-dense matter deposition was found in the basement membrane. Pathological diagnosis was MN stages I–II (**Figure 1**). Chest computed tomography (CT) was performed, and tumor markers, hepatitis B virus, hepatitis C virus, and human immunodeficiency virus were screened. The results of all of these tests were negative. There was no history of nonsteroidal anti-inflammatory drug use.

**Figure 2:**
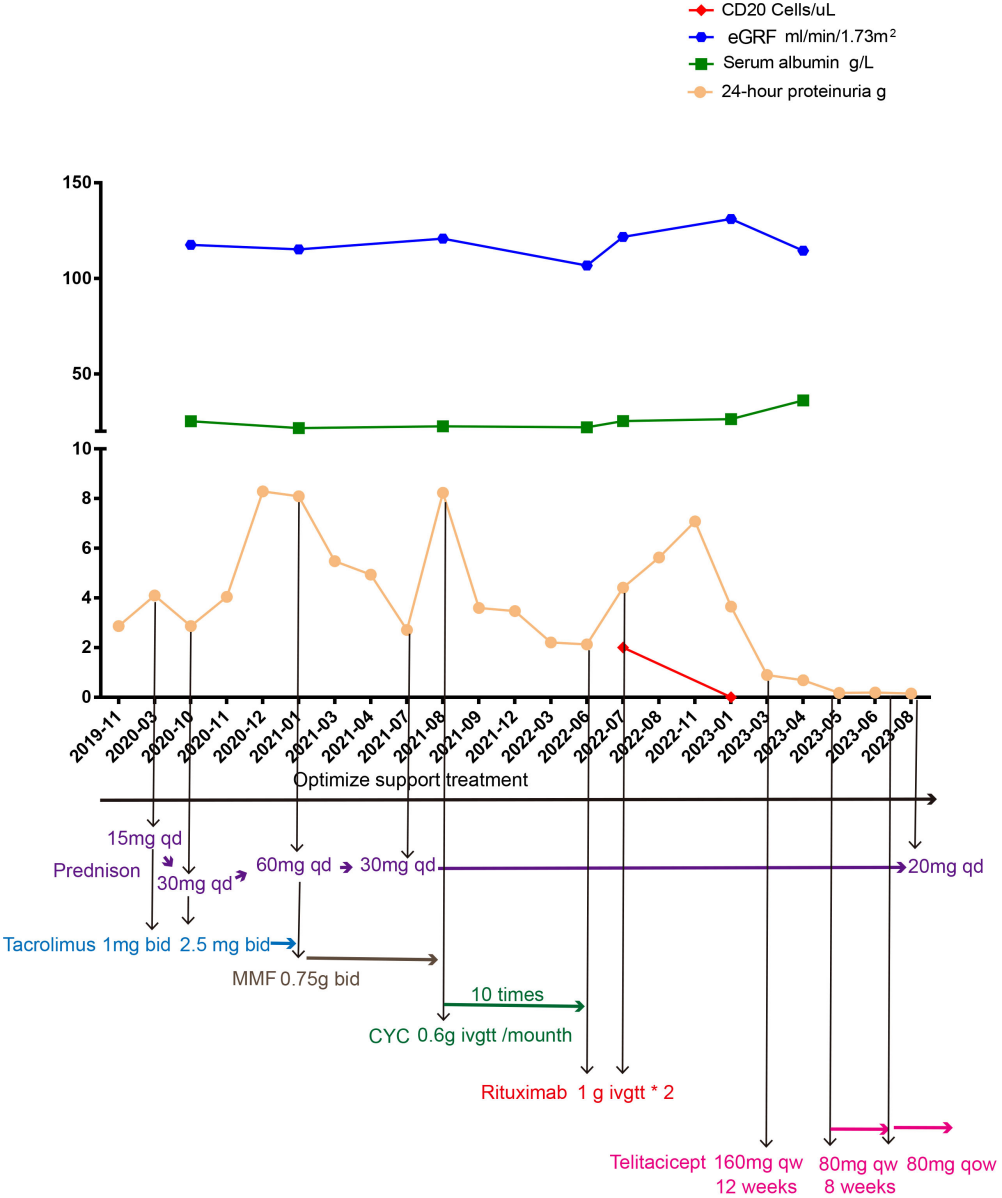
Overview of the treatment course. Cyclophosphamide (CYC), mycophenolate mofetil (MMF).

At first, clopidogrel 50 mg qd and valsartan 80 mg qd were given orally. In March 2020, 24h proteinuria (24h-P) was 4.1 g and prednisone 15 mg qd combined with tacrolimus 1 mg bid (gradually increased to 2.5 mg bid depending on the blood concentration) was added while continuing to optimize the supporting treatment scheme. In October 2020, 24h-P was 2.87 g, SA level was 25.2 g/L, serum creatinine (SC) concentration was 56.2 µmol/L, tacrolimus blood concentration was 6.5 ng/mL, and prednisone and tacrolimus were adjusted to 30 mg qd and 2.5 mg bid orally, respectively. In January 2021, the blood concentration of tacrolimus was 6.55 ng/mL, 24h-P was 8.09 g, SA level was 21.7 g/L, SC concentration was 59.3 µmol/L, and blood PLA2R and THSD7A were negative. Given that, at that time, the KDIGO 2021 clinical practice guidelines for the management of glomerular diseases had not yet been published, we recommended the patient to use rituximab or CYC. According to the KDIGO 2012 clinical practice guidelines and the evidence in the treatment of the Chinese population, mycophenolate mofetil was also introduced. Considering the economic burden and convenience of treatment, the patient insisted on using mycophenolate mofetil. We also fully reminded the patient that this prescription could pose a risk of treatment failure. The immunosuppression treatment plan was adjusted to include prednisone 60 mg qd in combination with mycophenolate mofetil 0.75 g bid orally, and prednisone was gradually reduced. In July 2021, prednisone was reduced to 30 mg qd in combination with mycophenolate mofetil 0.75 g bid. In August 2021, 24h-P was 8.23 g, SA was 22.6 g/L, SC was 52.8 µmol/L, and blood PLA2R and THSD7A were negative. This time, we recommended the patient to use rituximab or rituximab combined with tacrolimus. Due to the patient’s lack of confidence in tacrolimus and the high treatment and hospitalization costs associated with rituximab, CYC was ultimately chosen. The adjusted immunosuppressive treatment plan included prednisone 30 mg qd combined with CYC 0.6 g intravenous drip once a month. In May 2022, the patient received the 10th intravenous infusion of CYC 0.6 g. In June 2022, 24h-P was 2.13 g, SA was 22.1 g/L, SC was 60.6 µmol/L, and blood PLA2R and THSD7A were negative. We continued giving prednisone 30 mg qd orally to stop CYC, and rituximab 1.0 g intravenous drip was given. In July 2022, 24h-P was 4.41 g, SA was 25.4 g/L, SC was 60.6 µmol/L, CD20 count was 2 cells/µL, and blood PLA2R and THSD7A were negative. The second intravenous drip of rituximab 1.0 g was given, and after discharge, prednisone 30 mg qd was given orally. In January 2023, 24h-P was 3.65 g, SA was 26.4 g/L, SC was 43.3 µmol/L, CD20 count was 0 cells/µL, and blood PLA2R and THSD7A were negative. Although multiple immunosuppressive treatments were in use for more than 6 months, the examination results indicated a medium to high risk. Therefore, the patient was recommended to receive additional treatment with rituximab, and observation was continued. As the patient lost confidence in rituximab, a new treatment plan was expected. Considering that resistance was treated, referring to the treatment plan for treating resistance, rituximab has to be evaluated 3 months after use. If the treatment fails, CYC (already used) should be used, or the physicians should refer to the treatment experience of individual centers. Starting from 4 February 2023, 160 mg qw (12 weeks) telitacicept was administered, and the patient continued receiving 30 mg qd prednisone orally. In March 2023, 24h-P was 0.9 g. In April 2023, 24h-P was 0.69 g, SA was 36.2 g/L, and SC was 60.2 µmol/L. Starting from 29 April 2023, 80 mg qw telitacicept was administered, and in May 2023, 24h-P was 0.18 g. We reduced the telitacicept dosage to once every other week after 8 weeks of treatment with 80 mg once a week. Urinary protein remained in a complete remission state, and prednisone was reduced to 25 mg qd on 12 August 2023 (**Figure 2**).

## 3 Discussion

This is the first report of the use of telitacicept for the treatment of refractory MN, and complete remission was achieved. The KDIGO 2021 guidelines recommend that, for refractory MN patients who did not respond to rituximab or CYC, an expert center should be consulted and therapy such as bortezomib, anti-CD38 therapy, and belimumab should be considered (3). Another challenge in the treatment of this patient was that both blood PLA2R and THSD7A were negative, and the next prescription could not be established based on the antibody titer level. Therefore, in the patient’s treatment path, we focused on adjusting the prescription based on 24h-P and SA levels. When rituximab resistance appeared and CD20 count reached 0, we considered whether to continue using rituximab or replace it with another B-cell inhibitor. For this reason, we reviewed the pathogenesis of MN and the current mechanisms of action of related drugs to establish the next step of drug prescription.

**Figure 3:**
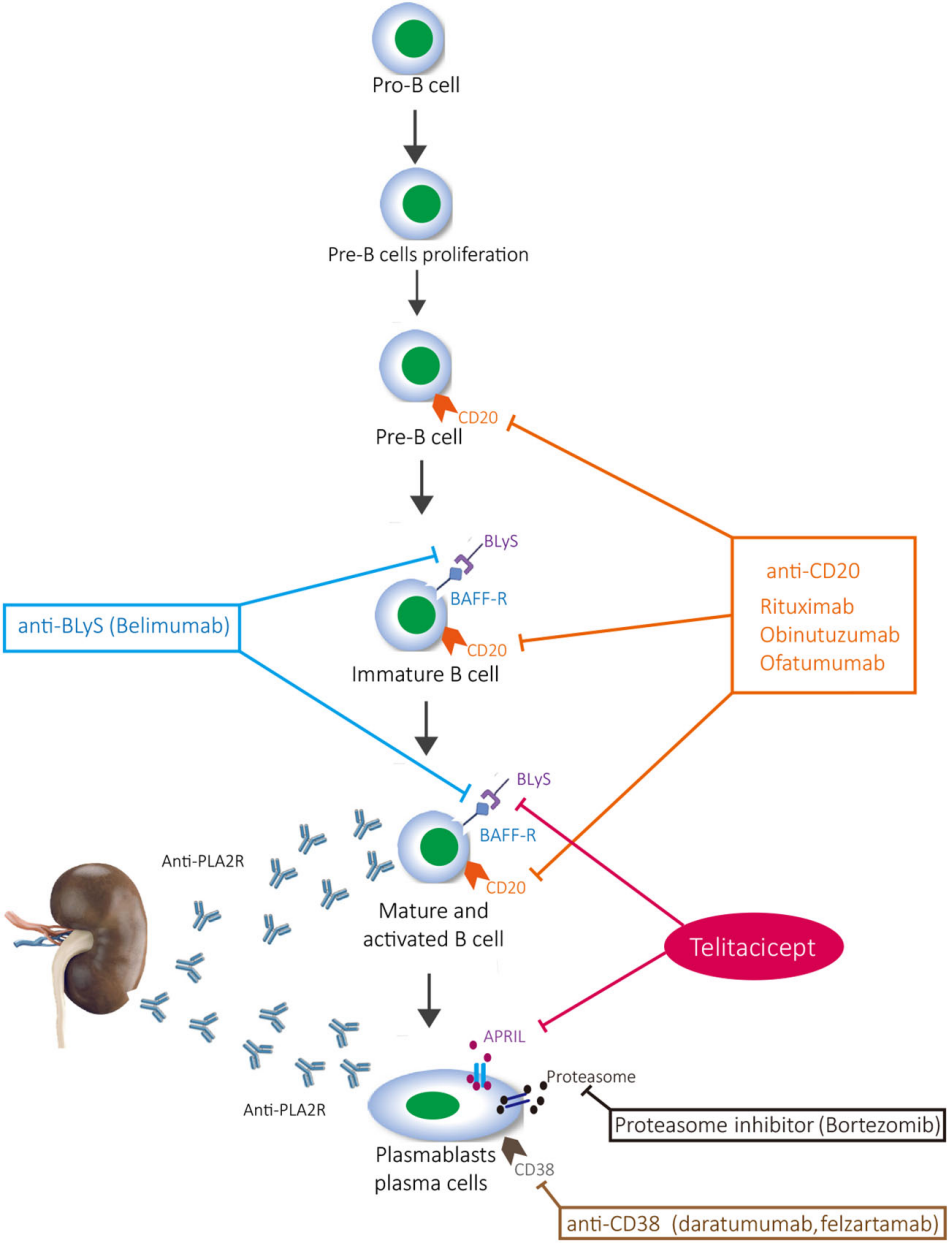
Maturation of B cells and drug inhibition targets.

The immune mechanism of MN involves the formation and deposition of immune complexes that contain immunoglobulins and complement, which induce podocyte damage and change the glomerular basement membrane, thereby leading to the development of proteinuria. Inflammation further develops into nephrotic syndrome and, if it continues to escalate, renal failure develops (8). Activated B cells and plasma cells are the main source of antibody secretion; when plasma cells are stimulated by antigens, they can produce a large number of antibodies (9). Rituximab is a classic drug for MN, and there is evidence that rituximab is superior to cyclosporine in maintaining proteinuria remission up to 24 months (10). Obinutuzumab, ofatumumab, and belimumab also target CD20-positive B cells, but they do not target plasma cells (11). Although proteasome inhibitors (bortezomib) and anti-CD38 (daratumab and felzartamab) inhibit plasma cells, they cannot inhibit CD20-positive B cells, which has certain limitations on the activation of B cells and the inhibition of immune response (12). Furthermore, it cannot be ignored that T cells are also involved in the activation of B cells (13). Although CYC and calcineurin inhibitors (tacrolimus and cyclosporin) inhibit T cells and their secreted cytokines, they lack effective inhibition of B cells, thus making it difficult to control the immune response from the source (14). The current clinical research results also confirm the limitations of the abovementioned drugs, that is, rituximab, CYC, and calcineurin inhibitors combined with glucocorticoid, to induce remission of proteinuria (15, 16) (**Figure 3**). The new type of B-cell-targeted therapy is not yet supported by evidence-based medicine and is only suggested in case reports (17–21).

Telitacicept is a protein that fuses a specific extracellular soluble part of transmembrane activator and calmodulin cyclin ligand interaction factor (TACI) with human IgG1 Fc segment through recombinant DNA technology. TACI receptor has strong affinity for B-lymphocyte stimulator (BLyS) and promotion-inducing ligand (APRIL). It blocks the interaction between BLyS and APRIL and their cell membrane receptors. By blocking BLyS, it inhibits the further development and maturation of immature B cells, helping to control the future development of disease. By blocking APRIL, it inhibits the differentiation of mature B cells into plasma cells, affects the secretion of autoantibodies by autoreactive plasma cells, better controls disease activity, and achieves multistage inhibition of B-cell maturation and differentiation. Meanwhile, due to the presence of TACI receptors on the surface of T cells, telitacicept also inhibits T-cell activation (22). At present, telitacicept is mainly used for the treatment of rheumatoid arthritis, systemic lupus erythematosus (SLE), multiple sclerosis, and IgA nephropathy (23). In children aged 5–18 years with active SLE, telitacicept combined with standard treatment significantly improves the response rate of refractory active SLE, reduces the dosage of glucocorticoid, and has a curative effect on lupus nephritis (24). The same effect has been demonstrated in the treatment of adult SLE (25). In a single-center, single-arm, and open-label study, eight patients with recurrent optic myelitis spectrum disorders were recruited in China. All of the patients underwent plasma exchange three times and then received telitacicept 240 mg per week for a total of 46 times. Two patients (25%) relapsed, and five patients (63%) still had no recurrence after 48 weeks of treatment (26). According to clinical empirical research on adult primary Sjogren’s syndrome (pSS), telitacicept has good clinical efficacy, tolerance, and safety in the treatment of pSS (27). In the treatment of primary glomerular disease, the current report mainly focused on a phase II clinical study of IgA nephropathy, which demonstrated that telitacicept could effectively reduce the level of urinary protein in patients (28). Recently, there have been case reports of the use of telitacicept for the treatment of refractory proliferative lupus nephritis (29) and minimal change disease (30). Based on the pathogenesis of autoimmune nephropathy and the mechanism of action of telitacicept, it is reasonable to believe that telitacicept has broad prospects in the treatment of autoimmune nephropathy (31). However, this case report has certain limitations. Due to the lack of evidence for the treatment of MN with telitacicept, the treatment regimen that we adopted needs further research and observation. It is not yet clear whether the patient’s condition will recur in the future and whether there will be adverse reactions. Further evaluation is needed to determine whether a multitarget inhibitor for B cells would be superior to single-target drugs. The therapeutic effect of telitacicept in MN deserves further attention.

## Data availability statement

The original contributions presented in the study are included in the article/supplementary material. Further inquiries can be directed to the corresponding author.

## Ethics statement

Written informed consent was obtained from the individual for the publication of any potentially identifiable images or data included in this article.

## Author contributions

LZ: Writing – original draft, Writing – review & editing. HJ: Methodology, Writing – review & editing. DW: Data curation, Writing – review & editing. YW: Writing – original draft, Writing – review & editing.
